# Supplementary material for: How Does Living in Temporary Accommodation and the COVID-19 Pandemic Impact under 5s’ Healthcare Access and Health Outcomes? A Qualitative Study of Key Professionals in a Socially and Ethnically Diverse and Deprived Area of London
Source: Int J Environ Res Public Health. 2023 Jan 11;20(2):1300. doi: 10.3390/ijerph20021300 (PMC9859054; doi:10.3390/ijerph20021300)
Supplement: Supplementary file 1 [file ijerph-20-01300-s001.zip › ijerph-2140213-supplementary.pdf]

Supplementary File S1 Interview Topic Guide

Interview guide for Professionals Study- Phase 3
Version 2: 16.02.21
Study title: The health of homeless children under-5 in temporary or insecure accommodation: barriers and facilitators to the access and utilization of health care services

Professionals:

- Health Professionals including Health Visitors
- Housing Experts
- Charities
- Key Informants (including Public Sector)

1. Can you start by telling me about your role at your organization: …………..?
   - Probe: Job Title, Self-identify profession, length at post, services provided, background

2. Broadly speaking, how do you define "homelessness" personally and by your profession?

3. In your service area, what do you see as the main issues affecting families with under 5s living in temporary accommodation in your area (Borough, Region)[adapted to interviewee and professional type]?

4. Do you/ Does your service experience any barriers in engaging/ working with families in temporary accommodation?

How easy is for you access the families , what are the main barriers you face, how has this been affected by COVID?
If yes, please briefly describe.
Probe: "Hard to reach"? Are there any cultural, language, geographical barriers? Have these barriers changed with COVID-19?

I'm now going to ask you a few questions related to health services:

5. What are your views on the experiences of families with under 5s accessing health services in your area?

   a. What do you think are the barriers to accessing health services for these families?
   b. What do you think are the facilitators or variables that help families to accessing health services or these families? (e.g. Charities, voluntary services)

Probe: 5 Components of Access- availability, affordability, accessibility, accommodation & acceptability

6. What are your views on whether under 5s are achieving healthy outcomes in your area (i.e. reaching milestones via Healthy Child Programme)?
   a. What do you think are the main barriers to them achieving healthy outcomes? (e.g. healthy weight, good nutrition, oral care, immunisation uptake, sleep hygiene, disability)

b. What about barriers on the individual and family level (e.g., race or ethnicity, immigration status [NRPF], and fear)?

c. What about on the systems level (e.g., poor access to medication, absence of care plan, and no insurance)?
d. What about on the neighbourhood/community level (e.g., transportation limitations and poor housing conditions)?

7. How does your service currently respond to these issues? (in Question 4)

Has this changed during COVID?
Probe: Policies? Services? Cross-sector partnerships? Following government guidelines? natural vs. built environment

I'm now going to ask you some questions about temporary accommodation

8. What do you think of the quality of temporary accommodation in your area?
    a. What aspects are well designed?
    b. What aspects are poorly designed?
    c. How might the quality of temporary accommodation impact the health of under5s and their ability to achieve optimal health outcomes?

9. Do you think these issues extend beyond your geographic area and across England? And in what way? Is it a national issue?

Probe: The number of children in temporary accommodation in England; health; quality of housing (issues discussed in interview above).
How do you think the COVID-19 pandemic has changed these issues?

I'd now like to describe existing policies and a way of designing interventions called co-production. Co-production is "an approach to decision-making and service design rather than a specific method. It stems from the recognition that if organisations are to deliver successful services, they must understand the needs of their users and engage them closely in the design and delivery of those services."
https://www.involve.org.uk/resources/methods/co-production

10. From a policy perspective, what do you think of the existing policies that are in place that support under 5s living in temporary accommodation?
    a. what recommendations do you have to address some of the barriers that you have described today?
    b. Are you familiar with the Homeless Reduction Act?
    c. What about Section 17, Children Act?
    d. What about the Housing Act?

What could we do better, are there any policy changes that are needed or should be revised?

Probe:
Homelessness Reduction Act (HRA)*-earlier intervention to reduce homelessness*
Section 17, Children Act*- ensure children are safeguarded and best cared for in their own families; acts a safety net for homeless families*

- *Examples: Councils are not required to report on families housed by children's services, so there is no central data to monitor whether the numbers of children in this group are increasing or what type of accommodation they are housed in.*
- *The regulations setting out the kind of accommodation homeless families should be housed in do not apply to families accommodated by children's services, so councils can decide what counts as suitable housing.*
    - *For example, there is no legal limit on the length of time a family can be housed in a B&B.*
- *Children's services lack expertise in housing, and some do not work closely with housing departments.*

Housing Act*- not all homeless families have a right to accommodation from the Housing Department or are eligible for accommodation under the Act such as:*

- *Families with* ***No Recourse to Public Funds***
- *"Families might also be judged to be* ***"intentionally homeless"*** *if they left their home when they could have stayed, if they failed to pay their rent despite it being judged affordable, or if they were evicted because of their behaviour." (Children's Commissioner (England) (2019))*

11. Based on the issues we've just described, can co-production be utilised as a tool to address them?

Probe:
Do you think families of under5s can play a role in co-designing and advising interventions, programmes, and policies that will impact them?
How can we better engage with families to address these issues?
How can we engage with other sectors and also policy makers?

12. Low- and Middle-Income Countries are experienced at delivering screening and early intervention programmes at scale, where there is economic restraint and low resources, a phenomenon which high-income countries are now experiencing. Examples include health camps, mobile health clinics and school-based interventions. Do you think this would be feasible or acceptable in England to implement such programmes to make sure that we can reach under5s who are experiencing homelessness to ensure that all under5s are reaching their milestones, getting their immunisations and attending their HCP screenings.

Prompt: Mobile health clinics have been utilised as a means to facilitate access to the most vulnerable and marginalised, and have been tested in HIC settings including the United States to deliver a range of services including urgent care and chronic disease management.

13. Do you have anything you would like to add that we haven't covered?

Supplementary File S2 Version 4 Codebook

Code	*Definition*	*Description*	*Qualification or Exclusion*	*Examples*	*Origin*
1. Background Information					
1.1 **Stakeholder group**	*Health Visitor<br>*Housing Expert<br>*Charity/Voluntary Sector<br>*Local Authority<br>*Children Centres	Occurs when interviewer asks about job title and organization<br><br>Biographical information of interviewee	Q: Organisation;<br>Profession;<br>Title of department	"I work for Newham On the ages 02 to 19 children's health"	Mixed-methods, cross-sector approach
1.2 Role at organisation	Biographical information	Occurs when interviewer asked about role at organization	Q: Job Title<br>Length at Post<br>Training<br>Experience	"I'm a specialist health visitor, it's all for children with complex need and the clinical team leader for the team. I've been doing. I've been working as in health visiting service, it's all for like this for 10 years now. And in this special role for five years"<br>"Housing, GPs, schools, um the Home Office cause my role now is is basically around immigration and supporting families to regulate their UK status."	Mixed-methods, cross-sector approach

Code	Definition	Description	Qualification or Exclusion	Examples	Origin
1.3 Service Provided	Services provided *Sum of all codes references in this section. Also when role is very broad for categories below.	When interviewee describes the types of service(s) they provide in general	Q: service descriptions<br>E: Not a challenge in providing the service; not changes in services during COVID<br>E: Not the title of service (1.1).	"My role covers mental health, housing and employment support So quite broad role."	Mixed-methods, cross-sector approach
1.3.1 Referrals	Referrals provided	Referral services and types of referrals by the professional; (ex. Charities, food banks, voucher programmes); including relevant signposting	Q: description of referrals; where referrals are made; providing vouchers for food banks, etc.; signposting	"So my role involves many aspects of this. It's supporting it's it's supporting with housing referring to shelter charity liaising with the charity."	Candidacy Framework; SEM
1.3.2 Needs and Development Assessment	Needs and Development Assessment	Professional carries out assessment in order to see how to best support under5 and family; also includes how to best promote health and development of the child at home; includes developmental reviews; can include risk assessments	Q: descriptions of assessment; types/names of assessments. Home visits; or reasons for assessment	"Yeah, so, so we basically ascertain which areas of need family are having issues with and then we will try and support them in these areas. So we have we've developed a assessment form. You may have heard of the traffic lights assessment."	Same; Healthy Child Programme; SEM
1.3.3 Toilet Training	Toilet training	Toilet training- based on off needs and development assessment	Q: descriptions of training	"toilet training. Yeah, it's basically gives us a little bit of structure to our assessments. So we offer toilet training we normally use what we used to do toilet training clinic."	Same

Code	Definition	Description	Qualification or Exclusion	Examples	Origin
1.3.4 Family Support	Family Support	Parents; siblings	Q: types of support services given to family; specific to parents and/or siblings	"All the other children under five are also supported by us. So if mum gives birth. We will support them with the new baby Well as a city when we're assessing the family's needs. We pick up these particular issues. We will try and do whatever we can to help support the family."	Same; Healthy Child Programe; SEM
1.3.5 Housing Advice	Housing Advice	Housing-related support or advice on housing options.	Q: Providing housing advice or professional writing a letter to housing office to get better housing.; finding better accommodation<br>E: Any mention of professionals (HP/HV)discussion of child or families specific needs in writing a letter gets coded under 5.2.2 Systems level facilitator mitigating barriers	"And so part of my job role essentially really is to support these families and by advising on advising them on their housing options and ensuring that We we assist to challenge bad housing."	SEM; health map for the local human habitat (built environment)
1.3.6 Prescribing	Prescribing medications and ointments	Professionals providing prescriptions	Q: must include "prescription" or "prescribing"	"And then yeah so like I said, you know, I was at the time I was a prescriber so I was able to you know if they needed and ointments environment saw creams, and things like that I could I could prescribe those from the small register that we were allowed to prescribe it on and trying to think what else."	HCP

Code	*Definition*	*Description*	*Qualification or Exclusion*	*Examples*	*Origin*
1.3.7 Immigration Assistance	Support with Immigration Status	Anything related to supporting families through immigration application process; Home Office	Q: Must be related to Immigration Application process and visas	"So, I work with children and families who in in my current role, who were deemed destitute and homeless. So, I assess them up with a single assessment at the single assessment, capturing their narrative. UM, but before I can do that, I need to get consent from the families in order to check, do conduct my checks with health, Housing, GPs, schools, um the Home Office 'cause my role now is basically around immigration and supporting families to regulate their UK status."	
2. Definitions of homelessness (noted in constant comparison when professional and personal definitions were stated as the same or there was overlap between definitions).					
2.1. Professional	Definitions of Homelessness	Interviewee asked to define homelessness professionally	Q: Provided definition	"And from the work I do, I've seen situations where families are put in a temporary accommodation and it last forever more than two, three years in a TA even though it should be short, short like intervention for people have housing problems, then it goes on indefinitely."	Scoping Review<br><br>Background Literature

Code	*Definition*	*Description*	*Qualification or Exclusion*	*Examples*	*Origin*
2.2 Personal	Definitions of Homelessness	Interviewee asked to define homelessness personally	Q: Provided definition	“Homelessness to me is it's a situation where families do not have a let me say permanent accommodation or maybe if they do have a place to reside, or if they are in like a short-term accommodation. That's like a temporary accommodation.”	Scoping Review<br><br>Background Literature
3. Greatest Challenges in Providing Services					
3. Professional Barriers	Challenges in providing services	Discussion on challenges in providing a service for/ engaging/ working with families in temporary accommodation<br>e.g., Types of barriers to engaging/working with TA families<br><br>*Barriers for the interviewee providing the service; this may go under a subtheme in system-level barriers later	Q: Provider Side (their experience)<br>*Any examples given<br>*Must be their perspective<br><br>E: How provider responds to barriers<br>Coded under 5.2.2	Q: “Yeah, language is a big barrier in Newham, generally.”<br><br>Q: “Yeah, there are lots of barriers because they don't have fixed address. So when you send them appointment letter you know by the time the appointment letter get there, they've moved And because there is nothing like redirection of mail. So definitely, they're going to miss that particular appointment.”	*Research Objective<br>*Research Question: On the other end, what are the challenges from the professional side to engaging/working with families in TA?<br><br>Examples: Helen Lester & Colin P Bradley (2001) Barriers to Primary Healthcare for the Homeless: The General Practitioner's Perspective, The European Journal of General Practice, 7:1,6-12, DOI: 10.3109/13814780109048777

Code	Definition	Description	Qualification or Exclusion	Examples	Origin
3.1 Language & Culture Barrier	Language & Cultural barriers between the provider and families	Language barriers between the provider and families. Cultural barriers that create tensions in uptake of certain services. Difficulties getting an interpreter	Q: Provider side<br>*Any examples given<br>*Can allude to them	"Absolutely. So that is one big barrier language. There is also other barriers cultural differences."	Same as above
3.2 Transient Lifestyle	Difficulties of reaching families because of lifestyle	Difficulties of reaching families because circumstances always changing.<br>*Includes how services are affected<br>* This includes continuity of care; families being registered in another borough; professionals being able to liaise with other health professionals regarding the families care; Professionals being notified about families' circumstances<br>*Families being invisible/dispersed	Q: Specific examples of when/why this happens; Professional's side<br>*How services are affected***<br>**How they are/are not responding<br>Challenges associated with this.<br>*Families being invisible/dispersed	"Very good to phone them and say, okay, you've come for this appointment. Remember to book your daughter's or your child's immunization with your GP. But if you can't find them and they are hard to reach. How are you going to remind them? So those are the issues that we face."<br><br>"I think geographically we hold an element of responsibility, but that there's a barrier in communication between I think with some of the remains Of the clinicians may experience barriers in terms of communication around notification of movements into and out of the borough in a timely manner."	Same as above<br>*This is a barrier on the individual and systems levels

Code	*Definition*	*Description*	*Qualification or Exclusion*	*Examples*	*Origin*
3.3 Lack of knowledge of child protection system	Difficulties of providing services when families don't know the system	Ditto; lack of knowledge of policies or navigation of services [Note: This code was merged with Code 3.1 during Constant Comparison]	Q: Provider perspectives; any examples given.; difficulties navigating the system.<br><br>E: unawareness of services goes under 3.8 where families don't know their services exist	"You know, you're trying to explain to them, even when you've got an interpreter, they still might not understand because it's not within the normal culture, the normal understanding. They just don't understand the system, you know."	Same
3.4 Systems; Housing availability	Lack of housing; Housing Office	Describes the lack of housing and how this impacts the providers being able to support families; if families are too preoccupied to follow their advice; Difficulties dealing with the Housing Office; Also includes how the professional feels when entering TA	Q: Provider perspectives; any anecdotes or specific examples.; can also include their feelings about it; descriptions about if families are not following their advice because they are too worried about housing- feelings about this.<br><br>Discussions about the difficulties of dealing with the Housing Office.	"You receive an email from new housing saying there's no houses out there for this family at the moment. There's nothing. He may have to wait a year even being on emergency"	Same as above

Code	Definition	Description	Qualification or Exclusion	Examples	Origin
3.5 Virtual Assessments	Providing services virtually to families and under5s; the challenges associated with it	Difficulties assessing child and families through virtual appointment; geographical limitations, COVID-restrictions; self-isolating or shielding; lack of digital access	Q: Provider point of view; what are the challenges for the provider i.e. is it difficult to provide an assessment without face-to-face? Difficulties not being able to provide service because of technical difficulties, therefore unable to hear families; many variables to facilitate.	"The, the geographical barriers, a big one. Because actually, if we want to see someone face to face. It's makes it quite difficult. So assessing someone is sometimes limited to over the phone or video call if they have that ability. And that obviously gets in the way of a full assessment. If it's needed."	Research Objective (ties Phases 3 and 4 together)
3.6 Workload and capacity	How services are stretched; workload and capacity	Includes staff sickness during COVID; ratio of patient to provider; appointment system; difficulties providing access; long waiting lists	Q: Discussions of how the service provided or professional capacity or workload is affected/changed, etc.<br>E: impact on the families and under5s; that goes under Systems-level barrier	"So included. I think the access to additional services has been massively stretched. So, you know, health, visiting teams are all now starting working remotely. Lots of teams have had sickness and so people accessing help and support are limited by the services that are available and those services are facing the same challenges as a whole system. Where staff sickness is a big issue and capacity is a big issue. And similarly, for us, I think."	Mixed-methods, cross-sector approach

Code	*Definition*	*Description*	*Qualification or Exclusion*	*Examples*	*Origin*
3.7 Mistrust	Mistrust of services	Families mistrust or suspicion including fear of mainstream services which act as a barrier; families not being forthcoming about their situation	Q: when the professional says it is a challenge for them including families not being forthcoming about their situation; also talks about how families have lost faith in services	"yeah so all of those I suppose I would add to that suspicion of sort of mainstream services, a lot of them will have had issues with Home Office, so they can be naturally suspicious of any kind of authority figure we will fall under that umbrella for a lot of them."	Scoping Review
3.8 Unawareness of Services	TA families of unawareness of their services	Provider describing that a barrier or challenge for them is that TA families don't know of their service; so getting them in the door/accessing the service; provider can also describe challenge about not knowing the families are there when they move into the borough	Q: Has to be a barrier/challenge from provider side when prompted; provider can also describe challenge about not knowing the families are there when they move into the borough<br>E: Barrier/Challenge from the families perspective which would go under Section 5	"But I think, I mean, I think, quite often the barrier is more about getting to the service. So, not knowing about the support that we can offer and quite often clients are more likely to be kind of referred and that tends to be then when they're more in touch with another service more due to like crisis or it might be social services they refer to us back like the key issue is the housing situation. So I think that's, that can be a barrier."	SEM
3.9 Short time in position	Professional's short time in their current position and/role	Professional's short time in their current position and/role; their ability to answer questions specific to U5TA population in Newham	Q: Must be the professional's current role; indication of short duration from their perspective.	"yeah so with Newham I, I haven't been there very long, and I know that we've got a very high number of families, but I haven't.<br>And I know a little bit about what we're doing in terms of the housing strategy to reduce those numbers but I haven't got as much experience of actually engagement"	Literature and Transcripts

Code	Definition	Description	Qualification or Exclusion	Examples	Origin
3.10 Lack of Strategies	Lack of Strategies or outreach models for professionals to reach families in TA; can include the invisibility factor of these families; can refer to gaps in services	Professional's description of the lack of outreach models or strategies for reaching families in TA especially compared to rough sleepers for example. Discussion of Invisibility of families can also be included in this discussion. Could also be Professional's discussion on how they individually strategize when working with families because of lacking strategies/models.	Q: Same as description; can also be coded with 3.2 Transient Lifestyle depending on content- if geography and dispersion of families is discussed, coded under both or 5.2.2.1 Policy Recommendations if there is a recommendation in how this should change; needs to be specific to U5TA population. *They may also talk about the gaps in services and/or how they commission services.	"It is harder when population groups are a bit more dispersed in terms of where they're being so i'm say that, in the sense of i'm comparing it with rough sleeping and you can have more. There's more outreach models that you'll know certain hostels that you can work closely with. There might be outreach street workers i'm not saying it in any way, this makes this that easier, but you've got greater strategies, I think, with families in temporary accommodation and that can be a bit harder. I think they're not necessarily as visible in terms of being seen as homeless."	SEM; Inductive from interview
4. *Views on COVID-19*					
4. COVID-19	COVID-19 changes (barriers and & facilitators)	Occurs when interviewee is asked about 1) their service changes during COVID 2) new and/or exacerbated barriers during COVID *Examples or Anecdotes can be coded here *Discussion on any changes in the barriers in providing service	Q: includes COVID in discussion -Can also be either provider or family experience -These are perceived barriers or observed barriers *barriers and facilitators coded together for COVID-19	"So for people that do not have English as their first language, then it's a big barrier because they don't understand what you're saying. And then for some parents who have lost their job because of COVID 19 you know i don't think tablet or phone will be the priority, the priority would be how to feed their family. So, if they don't have a tablet or a phone, how am I going to do a visual appointment with them? So that is not possible, so that is another barrier. And even if they have this phone or	Research Objective (ties Phases 3 and 4 together)

Code	Definition	Description	Qualification or Exclusion	Examples	Origin
		positive/negative, greater inequity	* Important to note the difference based on their professional experience Note: other barrier/facilitator codes can be coded with this COVID code to make it more granular	they have tablet, some of them, you know, IT is an issue."<br>"We are all getting, you know, learning and learning now because of COVID some of them, they don't have the apps that we are using You know as health professionals. So, and if they don't have English as their first language, there's no way we are going to communicate with them and then load this app."<br>"They don't get what we're saying. And some of them don't even have their phone, they don't have enough money, you know, to like buying data internet. Some people go to the library, you know, to check their emails, because they couldn't afford to get like a broadband at home. But if there is lockdown every, everywhere. There's no way they're going to, you know, get that service."	
5. Barriers and Facilitators under 5s experience achieving healthy outcomes and accessing health services<br><br>*Origin for whole section- Research Objective:*<br>*"To qualitatively explore how key professionals perceive the experiences of U5TA in accessing healthcare and optimising health outcomes as well their own challenges of providing services to the U5TA population."*<br><br>*5 Components of Access*<br>*O Availability- types of resources vs. needs of client*<br>*O Affordability- price of service or cost to access service (unable to pay)*<br>*O Accessibility- accounts for geographic, economic, transportation resources- measured in distance*<br>*O Accommodation- appointment systems, hours of operation, cultural or language barriers, digital divide*<br>*O Acceptability-client characteristics- age, social class, gender, ethnicity, immigration status, housing status*					

Code	Definition	Description	Qualification or Exclusion	Examples	Origin
5.1.1 Individual and family level barriers	Child Health<br>Parental Factors (age,<br>gender, hereditary factors,<br>ethnicity, family size, language, etc.<br>Includes Immigration Status (No Recourse to Public Funds)	Views or examples<br>*Child Health- (e.g. healthy weight, good nutrition, oral care, immunisation uptake, sleep hygiene, disability)<br>*Parental Factors- (e.g., race or ethnicity, immigration status, and fear, parent prioritization of health; language barriers, education; parental decisions on prioritizing<br>*parental beliefs on medicine and treating their child; property being lost/ feelings of unsettledness and not having place to call a home	Q: These are perceived barriers or observed barriers. Important to note the difference based on their professional experience	*Child Health. "But I can say that the majority of them, they have, they are achieving their milestones even through that. Some of them I have issue with their communications issue with the developmental delays, but there are a lot of system in place to support them, you know, when they have all those issues."<br>*Parental Factors. "They don't know. Some of them are they, I told you I've seen families in temporary accommodation for more than three years. So, they are not sure of when they are going to move them so that anxiety is there. There's is low self-esteem for parent. They are not happy. They're not comfortable, you know, with what they've got. They're not able to mix with other parents when even when they take their children to school because they're not proud of where they are living So it it up, it has a significant impact, not on the child alone, then even on the parent. Parents Well, cannot look after their children. So definitely it's…It's like it's flows down, you know, so that that is I mean that social effects is is no good for for the children that we work with."	Background-<br>Healthy Child Programme core requirements<br>4-5-6-approach<br>Scoping Review Findings<br>-Concept Map<br>-SEM (individual and interpersonal)<br>- health map for the local human habitat (people/lifestyle)

Code	Definition	Description	Qualification or Exclusion	Examples	Origin
5.1.1.1 Mental Health	Mental health-related	Parental mental health, child mental health; anxiety, depression, uncertainty; emotional well-being	Q: Parent, child, or siblings<br>• As a barriers<br>• As an outcome<br>Must specifically reference words from the description	“Then it comes to social you know days a lot of mental health issues. There are a lot of anxiety, uncertainty, because they put them in a, in a, maybe bread and breakfast, And decide you are you are temporarily rehoused there, for how long?”	Same
5.1.1.2 Transient Lifestyle	Instability; loss of property; no fixed address	Instability; loss of property; shame about situations/not wanting to interact with other children or parents; stigma; no fixed address; reasons for living in TA and/or homelessness including domestic violence, kicked out by landlord, immigration	Q: Individual/family level barriers; instability, no fixed address; same as description<br>Note: Difficulties registering with GP also under Systems;	“So just so many things, loss of property for parents moving from one accommodation to the other they might have maybe data vision broken, then when they move again.”	Same
5.1.1.3 Language & Culture	Language & Culture	Understanding and speaking the language; cultural differences; can also include illiteracy; not understanding a diagnosis or not willing to accept it (inductive)	Q: individually/family focused e.g. differences in cultural practices from health recommendations; can overlap with health care access in systems-level if those services are not accessible.	“There is always barriers because there's many families were very limited understanding of English language”	Same

Code	*Definition*	*Description*	*Qualification or Exclusion*	*Examples*	*Origin*
5.1.1.4 Immigration Status	Immigration Status	Right to Work; NRPF Status i.e. access to public funds; discrimination; no leave to remain status	Q: discussion of anything related to immigration and their status	"Yeah, we have some parent like that, due to immigration problems, you know, there are able to parent. I know you're able to work and if they can't work, then they might not even be able to meet the family needs."	Same
5.1.1.5 Financial Insecurity	Financial insecurity	Unemployment- no access to employment training or opportunities; food insecurity (e.g. food banks); inability to afford transport; types of benefits	Q: same as description; can include the challenge from the provider side<br>E: Right to work would go under 5.1.1.4. Immigration Status	"Traveling it's another issue of it, as you have said living. I mean, eating a balanced diet, healthy meal could be a challenge because they're not able to afford three square meal so they just eat any available food."	Same
5.1.1.6 Education Level and Knowledge	Education Level and Knowledge	Education level; includes resourceful and knowledge; "ignorance"; not knowing how the health system works	Q: Parent's resourcefulness; highest education level; health promotion and understanding	"I think it's, I think, is more like ignorance it's lack of knowledge rather than deliberately not wanting to access. Yes, poor education as well, probably."	Same
5.1.1.7 Competing Priorities	Competing Priorities	Discussion of families competing priorities; examples- food, clothing, and how that might impact the health of the child; housing	Q: Same as description; a&b scenario- family is more worried x, y than z<br>E: If parental mental health is mentioned exclusively, it would go under 5.1.1.1.	"They're just about to be evicted from their homes. For example, I have a family life that she's focusing was worrying about eviction from the house. So she's not going to be prioritizing maybe something further down the line like taking her child out to Children's center for some extra curricular activities when she's worried about being evicted."	Same

Code	Definition	Description	Qualification or Exclusion	Examples	Origin
5.1.1.8 Child Health	Child Health- overall health conditions and/or conditions	Includes the health, growth and development of the child. Mental health is not categorised- it is paired with parental mental health.; includes healthy practices; good oral health and nutrition; autism; includes milestones.	Q: Child health conditions and outcomes; healthy practices; can include the effect of poor health care access but must be coded with 5.2.1.2; includes milestones- if there is more than one barrier, make sure to code that one as well e.g. poor housing conditions<br>E: Mental health outcomes- that goes under parental mental health 5.1.1.1; any child health outcome that is directly attributed to a housing environment variable should be in 5.3.1.	"In temporary accommodation. Yeah, huge challenges. I mean, we were the third most of our second or third most apply right in London. We've got high levels of obesity and high levels of malnutrition."	-Concept Map<br>-Healthy Child Programme<br>-SEM
5.1.1.9 Family Structure	Family Structure- Single parent /Co-Habited Households	Descriptions of family structure whether single parent/co-habited households; multigenerational; can include family dynamics (e.g. family roles, provider, caretaker)	Q: Descriptions of family structure whether single parent/co-habited households; multigenerational<br>E: Overcrowding discussions	"The sort of the family dynamic in the household and causing antisocial behaviour and difficulty between the families is having a lot of stress on the child."	

Code	Definition	Description	Qualification or Exclusion	Examples	Origin
5.1.2 Individual and family level facilitators	(age,<br>gender, hereditary factors,<br>ethnicity, family size immigration status, lifestyle etc.	Primarily occurs when interviewee asked about the Perceived facilitators or variables that help families to access anything these families access for health needs	Q: could address parental factors, individual needs	"So I think, I think they're all [language, cultural differences] definitely barriers, but I think that we always try to sort of think of ways around them like we always have access to language line. So we always have interpreters that we can use over the phone, which makes a really big difference."	-Concept Map<br>-SEM (individual and interpersonal)<br>- health map for the local human habitat (people/lifestyle)
5.2.1 Systems-level barrier	Systems-level barrier; Systems (access to health services, policies on national and local levels regarding health care, housing, migration,<br>poverty, etc.)<br>Components of Access- availability, affordability, accessibility, accommodation & acceptability	Provided examples of systems barriers that impact health outcomes e.g., poor access to medication, absence of care plan, no insurance, policy, not having money to travel to health services; facilities being closed due to national policy so children's centres, community; lockdown; policies on<br>*not being able to register with GP because of no fixed address<br>*moving home*- top-down impact<br>*cultural differences affecting difficulty navigating the system	Q: These are perceived barriers or observed barriers. Important to note the difference based on their professional experience<br>Q: ID or description of policies; lack of familiarity if relevant; Opinions on existing policies when prompted<br>*Comments on current policies- how they work even if negative<br>*Comments on "moving home" anything that has to a policy relocating the family and child	"They are not able to assess health you know facilities easily because by the time they move from this address to another one. They automatically deregistered by the GP so for them to have another access to another GP, GP, they have to start all over. And before they finished up process, they are moved again."<br><br>"When they are you know about to become homeless if they, if there is a robust, you know, support for them.<br>I think that will be very good, because oftentimes when we have families that are about to become homeless and we contact social care they tell you to wait until the last day of eviction Which is like always late you know for for for majority of these families. At that point in time they've been evicted because they've been told to wait for wait until the last day. So, by the time they moved them. They just take them anywhere. And because those families they don't have anywhere to go to,	- Concept Map<br>-SEM (organizational and policy enabling)<br>-Scoping Review Findings Background literature-Healthy Child Programme core requirements 4-5-6-approach<br>*Examples of Components of Access https://www.healthanalytics.gatech.edu/focus-area/access<br>*Settings Framework

Code	*Definition*	*Description*	*Qualification or Exclusion*	*Examples*	*Origin*
		Professional's knowledge and familiarity with specific policies; especially able to give any examples that interviewer doesn't list<br><br>*Homelessness Reduction Act (HRA)<br>*Childrens Act<br>*Housing Act, etc.<br><br>*Professional's comments about a particular policy in place (e.g. Negative)		they just accept anything, you know any anywhere."	
5.2.1.1 Housing availability	Housing availability	How housing availability or lack thereof impacts the family; can include generalisations	Q: Specific examples of the consequences of the lack of housing; families being put in unsuitable housing or rehoused far away due to availability; it's okay if it overlaps with some of 5.3	"Because of the housing situation they are not able to go to school because they move from one address to another."	Same

Code	Definition	Description	Qualification or Exclusion	Examples	Origin
5.2.1.2 Health Services Access	Health Services Access	Barriers to accessing services; NHS; GP registration; therapy appointments; immunizations; communication between providers; can also include child-facing services; continuity of care; availability of materials in other languages or non-British English/layman terns	Q: Specific examples of the barriers to access; cost, distance; not having interpreters; not being able to register with GP or not registered with GP with x,y,z reasons; continuity of care; it's okay if it overlaps with other levels of the conceptual model	"They are not able to assess health you know facilities easily because by the time they move from this address to another one. They automatically deregistered by the GP."	Same
5.2.1.3 Policies	Policies	policies on homelessness, housing, education and school, immigration, safeguarding, social services; application cycles and procedures; TA living (restrictions); changes in policies or funding of programmes	Q: Provider's view and discussion on current policies; or lack of knowledge of current policies; examples of TA policies that could be barriers; this could also be a description of defunding of certain programmes which impacts families such as children's centres or early oral health programmes.<br><br>E: Recommendations	"And as I said, to I mentioned about education, because by the time the education they apply to the school before the application process is completed. They've moved So it affect their education it affect their learning it affect them in so so so many ways it affects children's behavior."	Same

Code	*Definition*	*Description*	*Qualification or Exclusion*	*Examples*	*Origin*
5.2.1.4 Digital Inaccessibility	Digital Inaccessibility	language, health materials online or just in English; number of applications or appointments need to be made online; unable to access wifi/internet for x,y,z including virtual appointments	Q: online materials; services access; electronic equipment; need to be tech savvy; unable to access wifi/ internet; some sort of digital or internet description by the profession<br><br>E: doesn't include my inference/theory as a reason	"Yeah, that is a big barrier as you have highlighted too, because as we said language barrier is there so many of these<br>An electronic or this equipment, So many information on the NHS website, they are like in Britain in English."	Same
5.2.2 Systems-level facilitator	Systems-level facilitator<br>Systems (**access** to **health services, policies** on **national and local levels** regarding health care, housing, migration, poverty, etc.)<br>Components of Access- availability, affordability, accessibility, accommodation & acceptability	Provided explanation of mitigating barriers e.g. accessing health services, achieving healthy outcomes even if brief<br>i.e. making referrals, using interpreters<br>*financial assistance<br>*this can include how their service facilitates<br><br>***Policy Recommendations**<br>***What needs to happen or the ideal**	Q: Any specific examples<br>*Important to note which examples of their service. Professional writing letters;<br>*Includes professional's ideas for policy recommendations<br>*Includes what they think is the ideal system or policy; or what needs to happen	"Yeah, language is a big barrier in Newham, generally. So both we've overcome that barrier is by using the Language Shop . So we book a language or an interpreter will want to do that kind of in visits or contact that kind of family and as you've said hard to reach. It's not because they don't want to be available, but because they are they are moving. You know, quickly. So that's why they become hard to reach."	-Concept Map<br>-SEM<br>(organizational and policy enabling)

Code	*Definition*	*Description*	*Qualification or Exclusion*	*Examples*	*Origin*
5.2.2.1 Policy Recommendations	Policy Recommendations	Provider's recommendations for policy change and/or intervention	Q: Recommendation; can be related to any of the aforementioned barriers previously discussed	"Like when they move from one borough to another if they could, if the GP GP in the whole new area is I don't know out that service agreement can work if they're not deregistered quickly"	Same
5.2.2.2 Mitigating Barriers	Mitigating Barriers	How the providers or relevant sectors mitigate the barriers; examples of support programmes available in different areas; how provider mitigates barriers- it could be helping families make GP appointments, etc.<br><br>Professionals writing letters to housing department if a problem with their housing e.g. GP says mould is causing a problem	Q: Existing programmes; examples; supporting services; example of a barriers and how it is mitigated.; Professional providing an example on how they mitigate a specific barrier; if this is a service too, it gets coded under Section 3 as well.<br><br>* HV and HP Writing letters for housing office.	"It's really to access triple P parenting program early start. It's called this organization is only based in New early start. So there will be offered currently they've been offered zoom a support sessions within a small group parent group."	Same
5.3.1 Neighbourhood/ community level barriers	Neighbourhood/ community level barriers<br><br>Housing Environment (social networks to green spaces to living conditions to geographical	Provided examples at this level that impact health outcomes:<br>Poor housing conditions, overcrowding,<br>Access to green space,<br>Physical Environment,<br>Social Network<br>-Views on quality of temporary accommodation	Q: These are perceived barriers or observed barriers<br><br>Important to note the difference based on their professional experience<br><br>*Can be specific examples of health	"Yeah, a lot of those accommodation there nothing to write [call] "home" about, you know, overcrowded poor condition. You see damp, mould on the wall. Some of them do have poor ventilation. You know, so there's a lot of things that are wrong with you know temporary accommodation."<br><br>"If you actually visit these families because we go there so many times,	-Concept Map<br>-SEM (community, housing)<br>- health map for the local human habitat<br>*Environmental-based factors- built environment (local economy, activities)<br>-Settings Framework<br>-Scoping Review Findings

Code	Definition	Description	Qualification or Exclusion	Examples	Origin
	distribution of resources.)	-personal experiences working in TA as a professional	outcomes caused by poor housing conditions	majority of them are concerned 95% of them are in poor housing environment. They're in poor accommodation You know, because it's like an emergency arrangement, so, they are like don't does the way I see it in bread and breakfast that is dirty and That are not suitable for families so you know properties."	- Citizen Science findings
5.3.1.1 Overcrowding	Overcrowding/ pollution	Discussions of overcrowding- number of people in TA or room size; also includes pollution and air quality even if it's caused by road pollution coming inside; number of people sharing a bedroom/rooms, etc.	Q: Anything related to overcrowding or lack of space i.e. not enough space to play or carry out physical therapy or follow any professional's recommendation as it relates to space; also can include pollution and air quality	"They dont have space to you know what smoke on the balcony or i'll go smoke outside for single mom. Families and they've been placed like in a room. You can give all the support, but what guarantees that they will follow those strategies yeah so that is lots of impact, you know it's having children that lives in temporary accommodation."	- Citizen Science findings & thematic analysis
5.3.1.2 Dampness and/or mould growth	Dampness and/or mould growth	Presence of dampness and/or mould growth in TA; can also include anecdotes about how family and child is affected; cases of asthma and types of infections	Q: discussions of dampness and mould in TA	"And also families living in poor housing so things like damp is a real issue and just living in housing in that's really not fit for purpose so sometimes you can go into housing where the conditions, are just appalling there's damp all over the walls. There's issues with heating and families, not be able to afford heating. yeah I think that's the main issues that i've come [across] so far."	- Citizen Science findings & thematic analysis

Code	Definition	Description	Qualification or Exclusion	Examples	Origin
5.3.1.3 Noise from Neighbours	Noise from Neighbours	Noise from neighbours and how that impacts the child and family	Q: can be noise experienced by the family or the family creating the noise which causes conflict with other residents.; can even be alluded to by discussing neighbours	"You know, when autistic children are unable to communicate verbally, so he does, a lot of them sort of banging ~~king~~. And makes a lot of noises. And so there is a lot of conflict between mother and child upstairs and the family downstairs and all of the services that this child had initially and sort of speech and language and, and all of these different groups have all been cut and and being confined into this small flat upstairs has been affecting his development, which is causing it can cause an obviously a negative impact on his well being, but also affecting The sort of the family dynamic in the household and causing antisocial behavior and difficulty between the families is having a lot of stress on the child."	Concept Map<br><br>-SEM<br><br>(community, housing)
5.3.1.4 Pest or Vermin	Pest/Vermin	Infestations	Q: anything related to infestations-pests/vermin	"So I would say, yeah, on the whole, lots of issues with things like damp, mold and pests as well. Quite often, people have mice issues or bed bugs."	- Citizen Science findings & thematic analysis

Code	Definition	Description	Qualification or Exclusion	Examples	Origin
5.3.1.5 Additional housing issues	Additional housing issues	Discussions of poor housing conditions and/or inadequate housing; sharing of kitchens or bathrooms; not being able to follow professional's instructions/recommendations because of housing environment	Q: any additional issues not covered above go here; examples of not being able to follow professional's instructions because of housing environment; or any general comments about the overall condition of housing.	"The main issues are around in temporary accommodation is the conditions of the properties, the accommodation they're living in, quite often and I have seen not only in this service, but I have seen properties that are in real disrepair."	- Citizen Science findings & thematic analysis
5.3.1.6 Lack of Social Capital-Support	Lack of social capital and support	Isolation from charities, families, friends, connection; difficulties moving into a new area and not knowing anyone; unable to socialize	Q: losing connections; same as description	"They're not able to mix with other parents when even when they take their children to school because they're not proud of where they are living, so it has a significant impact, not on the child alone, then even on the parent. Parents well, cannot look after their children. So definitely it's like it flows down, you know, so that is I mean that social effects is no good for the children that we work with."	SEM, concept map
5.3.1.7 Overall TA safety	Lack of safety provisions in TA/Overall TA Safety<br><br>**No safety provisions**	Lack of safety provision; steep stairs; windows, not baby/childproofed; floor that TA is on; also safety in general of the overall TA; family put in housing that is not appropriate for child's needs (e.g. wheelchair user and living on 3rd floor without a lift)	Q: any descriptions of housing regarding safety; could also be families not feeling safe	See text and Code **5.3.1**	SEM; citizen science findings, concept map

Code	Definition	Description	Qualification or Exclusion	Examples	Origin
5.3.2 Neighbourhood/ community level facilitator	Housing Environment (social networks to green spaces to living conditions to geographical distribution of resources.)	Quality of temporary accommodation in geographic area<br><br>Interviewee provided opinion/view/explanation on TA quality in their geographic area that they provide service in (either indirectly or directly)<br><br>Social network- what charities, religious are working at grass-roots level in community	Q: Anything related to housing environment (+), access to resources<br>e.g. housing bills covered- rent, heating, water; good quality housing<br>e.g. anything with religious groups, charities, food bank including the referral process<br>specific examples (e.g. Charities, voluntary services)<br>*Might have some overlap with 5.2.2.2.	"We could refer them to their some charity organization that able to support Families with no recourse funds and we have food bank. We are. We refer people to if they don't have enough, you know, to eat on them. They are few. I mean, there are some charity organizations are quite good in supporting families, you know, Who are, in that category."	Concept Map<br><br>SEM<br>(community, housing)<br>- health map for the local human habitat<br>*Environmental-based factors- built environment (local economy, activities)<br><br>Settings Framework<br>-Scoping Review Findings<br>- Citizen Science findings
6. Perceptions of barriers to access and achieving optimal health outcomes extending beyond their geographic area					
6. Perception of barriers beyond geographic area	Perceptions of barriers to access and achieving optimal health outcomes extending beyond their geographic area	As described in definition	Q: open-ended, yes/no responses, any statistical knowledge	"No, no, it's not. It's not. It's not in Newham alone. I think it across. I remember when I was training to become a health visitor. I wasn't in Newham I didn't train in Newham and the Properties. I've been to, you know, in those areas to they were appalling not into right to me. So it's not about Newham only. I think it's a general, you know, national problems."	Research Objective<br>*ties to Phase 3- Families Study- Quant data<br>*ties to Phase 1- scoping review findings and background literature<br>*Settings Framework
7. Views on Co-Production as way to address previous issues					

Code	Definition	Description	Qualification or Exclusion	Examples	Origin
7. Co-Production	Views of Co-Production (Role of families of under5s)	Professional's views of Co-Production Specifically do they think it is a good idea after being read the description: Co-production is "an approach to decision-making and service design rather than a specific method. It stems from the recognition that if organisations are to deliver successful services, they must understand the needs of their users and engage them closely in the design and delivery of those services."https://www.involve.org.uk/resources/methods/co-production	Q: open-ended<br>*can be an example they know of or co-production they are currently working on. Professional's familiarity with concept and/or ability to give examples<br>*Examples can be their own field examples from experience or outside. (Role of families of under5s in co designing…. Interventions, programmes, policies)	"Yeah, because the service users, their Voice must be heard. Yeah."	Research Objective<br>Background Literature<br>*Gelberg et al. (2004) Homeless Women's Suggestions for Improving Information, Education and Services<br>*Participatory approaches- Robert Chambers, 2008<br>*Citizen Science- Bonney et al., 2009<br>Phase 2- Citizen Science- participatory- methodology used to develop it<br>Phase 5- Recommendations<br>Definition: https://www.involve.org.uk/resources/methods/co-production<br>Supervisors
8. Reverse Innovation: application of LMICs in UK setting to deliver screening and early intervention programmes at scale					

Code	*Definition*	*Description*	*Qualification or Exclusion*	*Examples*	*Origin*
8. Reverse Innovation	Familiarity/Opinion<br><br>Examples from experience or knowledge in application	Interviewee explained that "Low- and Middle-Income Countries are experienced at delivering screening and early intervention programmes at scale, where there is economic restraint and low resources, a phenomenon which high-income countries are now experiencing. Examples include health camps, mobile health clinics and school-based interventions."<br>*Ask their opinion, feasibility, acceptability, familiarity with concept	Q: Anything on the discussion of Reverse Innovation and/ or<br><br>Professional giving any UK-based examples that they know that might fit into the category of reverse innovation	"Definitely, I think definitely for the mobile hubs like UM and traveling around particular areas and localities would be really useful to screen children under 5 UM specifically for those families who are living in temporary accommodation, I think that would be a good way of trying to. Identify what the main UM. Health issues are for that child for those families who are living in temporary accommodation. So definitely, They used to have them in the UK years ago and I think I saw a couple of them, it could be about three years ago when I used to live in Ilford. You know how like they have the mobile breast screenings pop-ups."	Research Objective- ties to Phase 5- making recommendations<br>*Settings Framework<br>*Scoping Review Findings<br>*Redlener, I., & Redlener, K. B. (1994). System-based mobile primary pediatric care for homeless children: the anatomy of a working program. Bulletin of the New York Academy of Medicine, 71(1), 49–57.<br>*Research Commentaries on COVID: https://scholars.direct/Articles/public-health/aphr-4-011.php?jid=public-health<br><br>https://www.thelancet.com/journals/lanpub/article/PIIS2468-2667(20)30080-3/fulltext<br><br>*Supervisors- Reverse Innovation
9. Other					

Code	*Definition*	*Description*	*Qualification or Exclusion*	*Examples*	*Origin*
9. Other	Other added information during interview	Anything else that interviewee adds (in last question) to conversation that can't be coded under the categories above	Q: Open-ended<br>E: No response, nothing to add from interviewee	**See text**	NA

Supplementary File S3 Framework Matrix for Systems-Level Theme

Exemplar Quotes (y-axis= Codes; x-axis= Professional Group)
I. Professional Barriers: A-E
II. U5TA and Families Barriers: F-K

Professional Group				
**Systems-Level Codes**	**Health Visitors (n=7)**	**Health Professionals (n=4)**	**Non-Profit (n=2)**	**Local Authority (n=3)**
**I. Professional Barriers**				
**A: 3.10 Lack of Strategies**	None	Add to the argument when they do in paperwork and things but most the most of the time I don't get involved because they've got other services, helping with you know, social workers and people like that. And it is a difficult life because we talk about it sometimes you know I deal with nutrition i'm supposed to sit there and do my diet therapy stuff with people. (HP4)	None	...in the sense of i'm comparing it with rough sleeping and you can have more there's more outreach models that you'll know certain hostels that you can work closely with. There might be outreach street workers i'm not saying it in any way, this makes this that easier, but you've got greater strategies, I think, with families in temporary accommodation and that can be a bit harder. I think they're not necessarily as visible in terms of being seen as homeless. And yeah, so I think there is something about how we commission services are probably thinking bit more from a mental health perspective, how we commission services and support. (LA3)

**B: 3.4 Systems- Lack of housing**	You receive an email from Newham housing saying there's no houses out there for this family at the moment. There's nothing. They may have to wait a year even being on emergency [list] which is not good. (HV1)	You know you're not getting on as a family, because you're on top of each other. You've got kids with all these additional needs, I mean I get in mums sitting there and in tears in front of me, and I just think, I just want a magic wand and make it all better and all the families at the moment are saying we've got problems with our housing it's not right it's not right. It's just like to know what to do you know. How hard. (HP4)	None	And the third thing was the the really difficult nature of being in temporary accommodation in London in terms of the acute shortage of affordable housing how that was making different boroughs bid with other boroughs.(LA3)
**C: 3.5 Virtual Assessments**	So, again it's your assessment is heavily based on the testament of the parents. Of course, in the face to face, you were able to have a visual demonstration. So it has made it a bit more difficult to to assess. But then again we always have a fail safe, so we do have options to bring them in to clinic. (HV4)	So yeah it's something we're still battling with and again, you know with we work a lot with interpreters as well and that's been quite tricky online to to facilitate that and use of it and in terms of yet making sure that we're hearing our families and giving the service to them in the best possible way for them to be able to understand so and yeah it's still so that's a big issue as well.(HP3)	And for instance, you know, we would usually go and do home visits and we can assess people's needs that way and and sort of get beyond to the root of any underlying issues and identify any sort of more urgent issues that they may not necessarily approach us with but I guess that's we can't, we can't make those judgments. Now, because everything is is digital. (NP2)	None
**D: 3.6 Workload and capacity**	None	That will make a huge difference actually because the all the health visitors are understaffed in the borough, like everyone else's. But their clinics are too fully booked. They haven't got enough time to do enough home visits, which I think is crucial, particularly in temporary accommodation that they I think they need to lay eyes on the situation and being able to visit regularly enough to monitor typically where they've got concerns about neglect. And I think with the with the amount of health visitors [and] mental health services we've got in the borough that's just not possible at the moment. (HP2)	Because I think before like quite [often] clients that I work with, quite often see the same health visitor like every week or something at the Children's centre because that's where he goes, so they just happened to kind of see them. But now it's kind of relying on that client or that health visitor to go out of their way to have a call or visit which obviously the health visitor has just to do to the best ability, but they have to see so many different families that maybe families those that they need support but who don't know how to reach out for it. (NP1)	None

**E: 3.9 Short time in position**	So I recently started my role [as a specialist health visitor] so i've only started about six months ago, so i'm still finding my feet.<br>But prior to this, I was a generic health visitor. (HV6)		Ah, so I should say that I'm actually new to the Newham service. So I've only been working within the service for the last two and a half months, which is when we've been in the second lockdown and a lot of my families have already been introduced to services, but their services have been cut because of the current climate.(NP2)	Yeah so with Newham, I I haven't been there very long, and I know that we've got a very high number of families, but I haven't and I know a little bit about what we're doing in terms of the housing strategy to reduce those numbers but I haven't got as much experience of actually engagement. (LA3)
**II. U5TA and Families Barriers**				
**F: 5.2.1.1 Housing availability**	You know, because some families, washing machine is not in working order they don't have proper you know and like I said before, some families, they live in their boxes because they sometime tell them oh you're going to be here for three months, six months and they're waiting to be moved forever and you've got families that they're really they've been living in accommodation for ages, some of them up to eight [years] you know so they're just sitting there waiting. Obviously, the demand is more in London. The demand for to live in London is more...(HV7)	It just feels like they're put somewhere because they have to be somewhere, but it does feel like that it just isn't appropriate, but then the idea is it's supposed to be temporary isn't it, but then it seems like these families are staying there for a long time. And then they having to bid so having to bid for new places and again that in itself is a struggle, you know. (HP4)	I wouldn't imagine that temporary accommodation in areas outside of big cities would have so much of the same issue, simply because there is more stock and to be able to provide long term housing, whereas the issue here is that we don't have long-term housing stock so any accommodation is accommodation and it will have to do. (NP2)	Families who have been displaced from where they originally were living in London and wanting to remain in London and then being placed out of London. Some families we have in Bradford, Leeds and you know, for them to to actually accept those properties, we've really got to promote it like a fresh start for them because a lot of them they're they're struggling in London and with the health issues. (LA1)
**G: 5.2.1.2 Health Services Access**	...because they you find that that they either missed things like immunization because they just moving from places to place so it's very difficult, it's very difficult for them to register with the GP also because they might not have a permanent place and sometimes all the the GPs are very difficult if you don't have any	I can't imagine it's helped because you know well, when I'm thinking about it has made a huge impact actually thinking about it because they're not getting, I've got lots of families who want to go to dentists and there's a specialist dentist service, but they can't get to them. Health visitors aren't going out, yeah there's lots thinking about it. There's a lot that's changed for COVID because if we've all	But it was so difficult. We couldn't do it. So that was how she would just do that. But obviously now that's not necessarily a possibility to just walk into the GP surgery. [during pandemic](NP1)	For the families that I work with, quite often, they've been turned away because they don't have any status in the UK, so quite often I will just write them a letter on letter headed paper requesting that this family are registered as new patients to their service, so their local GP. Because we move a lot of families quite far out of London,

	permanent address with them to register you.(HV5)	shut down, although we didn't, this is what I don't get, I still carried on doing, I did a lot of stuff with video intermittenly for a while, but we opened clinics up again. Not quite sure why everyone else hasn't. You know even that GPs and things like what's happening, so I do I do worry that you know they're not they haven't got the same access they would have before COVID because there just isn't the appointments, that we're, everyone's really behind. (HP4)		so they need to have a local GP and what we're finding is a lot of the time those same families are maintaining their GP where they used to live as opposed to where they live now, which could be somewhere quite far in Essex for instance. (LA1)
**H: 5.2.1.3 Policies**	I don't know whether there is any policy that is there for under fives living in temporary accommodation, because if there is then the policy is not, it's not working and it's not effective because as far as I know, with or without a policy, is still the same. The only thing I know with the policy is because you're supposed to care for to make sure the child receive equal care as someone who is not in a temporary accommodation...(HV5)	Honestly I don't really know what they are at all [laughs]. (HP2)	Because of, particularly in cases where it's unsuitable accommodation. It can be really difficult to get the local authority to accept that clients are homeless. But I would argue that they are due to the circumstances that they're living in and the impact is having on them.(NP1)	But the one that I did read was in relation to the shared accommodation for children and that shouldn't have no more than a family of four living in a family of four two sets of families living in one four-bedroom property, which I thought was actually quite fair because there in those properties there has to be 2 toilets. There has to be 2 bathrooms and you share the kitchen, which is usually a a decent size kitchen.(LA1)

**I: 5.2.1.4 Digital Inaccessibility**	I would say technology technology is a barrier, unfortunately, because of COVID the system if I say, for example, system of registration registration for GP has changed for everything, because we moved remotely and then that would require that you have use of wi fi Internet and also a basic grasp of of using the Internet so sometimes the parents might not have access to these tools, like the Internet If they're moving around so often and unable to get an Internet connection But then, but then unfortunately there's no there's no compensation for that they have the GP services they don't provide an alternative, so if you can't register online, then you can't register so, then that would put a barrier for that family accessing health services. (HV4)	I think we, we all assumed that most families did but it's still something that yeah not they all have especially if they're in temporary accommodation. I think you know things aren't always set up and because they don't know how long we're going to be there, or so yeah it's something we're still battling with and again, you know we work a lot with interpreters as well. And that's been quite tricky online to to facilitate that and use of it and in terms of yet making sure that we're hearing our families and giving the service to them in the best possible way for them to be able to understand so and yeah it's still so that's a big issue as well. (HP3)	But our main way of communicating with them moving forward to deal with their case would be via email and telephone and it's not very often that you will get<br>Good correspondence back and because it's just not the way that they are used to dealing with things. (NP2)	None

**J: 5.2.2.1 Policy Recommendations**	The only thing I can say is children section 17 children with additional needs they have rights have rights to live in a more<br>Clean warm, comfortable, safe environment like all other children.(HV1)	I guess as much as possible to think of the family holistically and what's best for them, and in terms of looking towards permanent accommodation, making sure that it's safe and that the children can thrive in the environment and what would be from a therapist perspective and yeah and as much as possible, you know, making sure that specifically if the children have got and special education needs that we're kind of even if they're in a pre verbal, listening to the voice of the child and as much as possible, giving them that time and preparation for that change, and they are going to be moving elsewhere, so you know as much as possible kind of giving the family notice and making sure they're happy with the arrangement before maybe for the for the child and visit the accommodation beforehand with kind of that transition.(HV3)	It needs to be something where there's clearer guidance, a bit more like say like the fitness for human habitation where that's more around like what what is deemed acceptable and what isn't. But I think there still needs to be something specific to temporary accommodation...(NP1)	And it's very much like know people goes to children's centers because they're local, but when you're talking about anything more than a mile out it's much harder and I think that that speaks a lot to the health and the inequalities agenda where we know that if you're going to reduce health and qualities and improve health outcomes for those most as socially disadvantaged, you need to make things easy and you need to make services really work to support them and to and to work with basic solutions like food provision better housing ...(LA3)
**K: 5.2.2.2 Mitigating Barriers**	You know professionals coming together, meeting, teamwork, recommendations being proactive with the Newham council housing. Eventually, we got the father, it's just a father and the child and is no mother involved. The father and child they've got emergency priority at the moment because they're staying a great grandfather's house. But there's no properties available to give the family, even if they're on the highest priority which this families on there's nothing out there at the moment to meet that child's needs. It has to be disabled access ground floor disabled access; it has to be suitable to the child's disability. So, it has to be in certain aspects, be able to use the bathroom. Adaptations .(HV1)	...workshops I think lots of things have gone online recently so trainings and workshops online, so I think that has really helped families as well if if they aren't able get to the centre but if they have moved and want to still access those services and so that's been really helpful and and yeah we do work quite closely with the Scope the sleep support charity and that's kind of not necessarily related but, and you know if the housing issues can be an<br>impact obviously a lot on on children's sleep, and so we kind of work quite closely with those with that sector as well, and I know there are some other kind of organizations to support parents Newham's SENDIASS, a parent forum.(HP3)	Quite often, the Council will say that they haven't been able to contact people for various reasons, but it's often because they might not be using an interpreter or might not be sort of finding out more about this client circumstances and the reasons why it's difficult for them to communicate. Like, I can say quite a lot of our clients have additional support needs so can have quite bad mental health or substance use. I think we really sort of go that extra mile to contact people and to rebook missed appointments and those types of things. I think [that] helps people to kind of stay engaged with the service a bit more.(NP1)	I mean we're doing interesting work in Newham looking at this homeless hostel that we've got some money to build and looking at service design and i'm not sure how much it would think about this in relation to families in temporary accommodation, but we're trying to think about building independence in that model.(LA3)

Supplementary File S4 Frequency with which themes were discussed by professional group: n and %

Code	Health Visitor (*n=7*)	%	Health Professional (*n=4*)	%	Non-Profit Sector (*n=2*)	%	Local Authority (*n=3*)	%	Total (n=16)	%
**1.1 Stakeholder group**	7	100.00	4	100.00	2	100.00	3	100.00	16	100.00
**1.2 Role at organisations**	7	100.00	4	100.00	2	100.00	3	100.00	16	100.00
**1.3 Service Provided**										
**1.3.1 Referrals**	6	85.71	3	75.00	1	50.00	0	0.00	10	62.50
**1.3.2 Needs and Development Assessment**	5	71.43	1	25.00	2	100.00	2	66.67	10	62.50
**1.3.3 Toilet Training**	2	28.57	0	0.00	0	0.00	0	0.00	2	12.50
**1.3.4 Family Support**	2	28.57	3	75.00	2	100.00	0	0.00	7	43.75
**1.3.5 Housing Advice**	0	0.00	0	0.00	1	50.00	1	33.33	2	12.50
**1.3.6 Prescribing**	0	0.00	1	25.00	0	0.00	0	0.00	1	6.25
**1.3.7 Immigration Assistance**	0	0.00	0	0.00	0	0.00	1	33.33	1	6.25
**2. Definitions of Homelessness**										
**2.1 Professional Definition of Homelessness**	6	85.71	3	75.00	2	100.00	3	100.00	14	87.50
**2.2 Personal Definition of Homelessness**	7	100.00	4	100.00	2	100.00	3	100.00	16	100.00
**3. Professional Barriers**										
**3.1 Language & Culture Barrier**	7	100.00	3	75.00	2	100.00	0	0.00	12	75.00
**3.10 Lack of Strategies**	0	0.00	1	25.00	0	0.00	1	33.33	2	12.50
**3.2 Transient Lifestyle**	4	57.14	3	75.00	0	0.00	1	33.33	8	50.00
**3.4 Systems- Lack of housing**	4	57.14	2	50.00	0	0.00	1	33.33	7	43.75
**3.5 Virtual Assessments**	4	57.14	1	25.00	1	50.00	0	0.00	6	37.50
**3.6 Workload and capacity**	0	0.00	2	50.00	1	50.00	0	0.00	3	18.75
**3.7 Mistrust**	2	28.57	2	50.00	0	0.00	2	66.67	6	37.50
**3.8 Unawareness of Service**	1	14.29	0	0.00	2	100.00	0	0.00	3	18.75
**3.9 Short time in position**	1	14.29	0	0.00	1	50.00	1	33.33	3	18.75

**4. COVID-19**	7	100.00	4	100.00	2	100.00	3	100.00	16	100.00
**5.1.1 Individual & Family-level Barriers**										
**5.1.1.1 Mental Health**	5	71.43	3	75.00	2	100.00	1	33.33	11	68.75
**5.1.1.2 Transient Lifestyle**	4	57.14	2	50.00	2	100.00	1	33.33	9	56.25
**5.1.1.3 Language & Culture**	6	85.71	2	50.00	1	50.00	1	33.33	10	62.50
**5.1.1.4 Immigration Status**	5	71.43	2	50.00	0	0.00	2	66.67	9	56.25
**5.1.1.5 Financial Insecurity**	3	42.86	2	50.00	1	50.00	3	100.00	9	56.25
**5.1.1.6 Education Level and Knowledge**	1	14.29	0	0.00	0	0.00	0	0.00	1	6.25
**5.1.1.7 Competing Priorities**	4	57.14	2	50.00	1	50.00	0	0.00	7	43.75
**5.1.1.8 Child Health Outcomes**	7	100.00	4	100.00	2	100.00	2	66.67	15	93.75
**5.1.1.9 Family Structure-Single parent/Co-Habited Households**	1	14.29	1	25.00	2	100.00	0	0.00	4	25.00
**5.1.2 Individual& family-level facilitators**	0	0.00	0	0.00	0	0.00	0	0.00	0	0.00
**5.2.1 Systems-level barrier**										
**5.2.1.1 Housing availability**	4	57.14	2	50.00	1	50.00	3	100.00	10	62.50
**5.2.1.2 Health Services Access**	7	100.00	4	100.00	2	100.00	3	100.00	16	100.00
**5.2.1.3 Policies**	7	100.00	4	100.00	2	100.00	3	100.00	16	100.00
**5.2.1.4 Digital Inaccessibility**	4	57.14	1	25.00	2	100.00	0	0.00	7	43.75
**5.2.2 Systems-level facilitator**		0.00		0.00		0.00		0.00	0	0.00
**5.2.2.1 Policy Recommendations**	7	100.00	4	100.00	2	100.00	3	100.00	16	100.00
**5.2.2.2 Mitigating Barriers**	7	100.00	4	100.00	2	100.00	3	100.00	16	100.00
**5.3.1 Neighbourhood & Community -Level Barriers**										
**5.3.1.1 Overcrowding**	7	100.00	4	100.00	2	100.00	1	33.33	14	87.50
**5.3.1.2 Dampness and/or Mould growth**	4	57.14	4	100.00	2	100.00	1	33.33	11	68.75

**5.3.1.3 Noise from Neighbours**	0	0.00	2	50.00	1	50.00	0	0.00	3	18.75
**5.3.1.4 Pest or Vermin**	2	28.57	0	0.00	1	50.00	1	33.33	4	25.00
**5.3.1.5 Additional housing issues**	7	100.00	4	100.00	2	100.00	3	100.00	16	100.00
**5.3.1.6 Lack of Social Capital-Support**	3	42.86	0	0.00	1	50.00	1	33.33	5	31.25
**5.3.1.7 No safety provisions (OVERALL TA SAFETY)**	3	42.86	2	50.00	1	50.00	1	33.33	7	43.75
**5.3.2 Neighbourhood & Community-Level Facilitators**	4	57.14	2	50.00	1	50.00	1	33.33	8	50.00
**6. Perception of barriers beyond geographic area**	6	85.71	3	75.00	1	50.00	3	100.00	13	81.25
**7. Co-Production**	7	100.00	4	100.00	2	100.00	3	100.00	16	100.00
**8. Reverse Innovation**	3	42.86	4	100.00	2	100.00	3	100.00	12	75.00
**9. Other**	2	28.57	1	25.00	0	0.00	2	66.67	5	31.25
**TOTAL**	192		108		61		70		431	
